# Giant Cell Arteritis Presenting With Ocular Symptoms: Clinical Characteristics and Multimodal Imaging in a Chinese Case Series

**DOI:** 10.3389/fmed.2022.885463

**Published:** 2022-06-20

**Authors:** Qian Chen, Weimin Chen, Chaoyi Feng, Deshan Gong, Jiong Zhang, Yingwen Bi, Ping Sun, Xinghuai Sun, Guohong Tian

**Affiliations:** ^1^Department of Ophthalmology, Eye Ear Nose and Throat Hospital of Fudan University, Shanghai, China; ^2^Department of Neurology, Shanghai Deji Hospital, Shanghai, China; ^3^Department of Neurosurgery, Shanghai Deji Hospital, Shanghai, China; ^4^Department of Rheumatology, Huashan Hospital, Fudan University, Shanghai, China; ^5^State Key Laboratory of Medical Neurobiology, Institutes of Brain Science, Fudan University, Shanghai, China

**Keywords:** giant cell arteritis (GCA), anterior ischemic optic neuropathy (AION), arteritic anterior ischemic optic neuropathy, color duplex ultrasonography, superficial temporal artery biopsy

## Abstract

**Purpose:**

To evaluate demographic and clinical characteristics of a Chinese population with giant cell arteritis using multimodal imaging focusing on ophthalmic examinations.

**Design:**

Retrospective observational case series.

**Materials and Methods:**

In the neuro-ophthalmology division of the Eye, Ear, Nose, and Throat Hospital, Shanghai, we evaluated the demographic and clinical characteristics of patients diagnosed with giant cell arteritis between January 2016 and June 2021. Results of routine ophthalmic examinations including fundus examination, optical coherence tomography, color duplex ultrasonography of ocular and superficial temporal arteries, orbital magnetic resonance imaging, and superficial temporal artery biopsy were evaluated.

**Results:**

A total of 15 patients (22 eyes; ten male and five female) were evaluated with a mean age of 77.0 ± 8.5 years. Among them, seven had bilateral involvement that occurred simultaneously or sequentially. Twelve patients presented with arteritic anterior ischemic optic neuropathy, two with arteritic anterior ischemic optic neuropathy combined with cilioretinal artery occlusion, and one with cotton-wool spots. In acute stages of optic neuropathy and retinopathy, optical coherence tomography revealed optic disc edema, thickening of the inner retinal nerve fiber layer and ganglion cell layer, and loss of layer structure. In late stages, optical coherence tomography revealed diffuse atrophy of the inner retina. The “halo” sign was observed in 12 patients in the superficial temporal artery ultrasound, and seven out of eight patients who underwent biopsy demonstrated classic giant cell arteritis pathological changes. Most patients having poor visual acuity but ability to perceive light; 10/22 eyes had permanent vision loss.

**Conclusion:**

Although rare in Asians, giant cell arteritis may be underdiagnosed among elderly Chinese patients presenting with anterior ischemic optic neuropathy. Non-invasive superficial temporal artery ultrasound detecting inflammatory thickening of the intima as the “halo” sign combined with routine elevated erythrocyte sedimentation rate and C-reactive protein may be helpful in diagnosing patients with a high probability of having giant cell arteritis.

## Introduction

Giant cell arteritis (GCA) is a type of systemic vasculitis that mainly affects medium-sized and large arteries. The incidence of GCA in Asians is 20 times less than that in Caucasians (1). Although rare, an increasing number of Asian GCA cases, with typical clinical features that are commonly observed among Caucasians, has been reported (2–6); however, only one nationwide survey has addressed that the prevalence of GCA in patients aged > 50 years in 1997 was 1.47 per 100,000 persons in Japan (7).

In 2018, we reported the case of a Chinese patient with biopsy-proven GCA, presenting with simultaneous anterior ischemic optic neuropathy (AION) with no light perception (6). To our knowledge, this is the first Chinese case report on the ophthalmic involvement in this disease. In the subsequent years, >15 patients presenting with AION and profound visual loss accompanied with headache have been diagnosed in our neuro-ophthalmology clinic, which indicates that GCA might be underdiagnosed among elderly Asian patients. The permanent visual loss associated with this neuro-ophthalmic condition requires careful vigilance.

In this case series, we utilized multimodal imaging techniques, especially non-invasive color duplex ultrasonography (CDUS), in elderly patients with ocular artery ischemic conditions who were at a high risk of GCA and for whom biopsy was not straightforward or available.

## Materials and Methods

### Patients

Between January 2016 and June 2021, patients with newly diagnosed GCA were admitted to the neuro-ophthalmology division of the Eye, Ear, Nose, and Throat Hospital in Shanghai, China.

The criteria for diagnosing GCA were as follows (8): (1) age of onset, >50 years; (2) new onset headache; (3) temporal artery abnormality on examination (tenderness or reduced pulsation); (4) elevated erythrocyte sedimentation rate (ESR) (> 50 mm/h); and (5) abnormal temporal artery biopsy, revealing necrotizing vasculitis with predominant mononuclear cell infiltration or granulomatous inflammation. Patients who presented with at least three of these five criteria were enrolled.

Exclusion criteria included: (1) polyarteritis nodosa or other conditions suspicious of systemic vasculitis (9), (2) inability to cooperate for examination to diagnose GCA, (3) incomplete clinical data, and (4) refusal to sign the consent form.

### Demographic Data and Ophthalmic Examinations

Data regarding age, sex, lateral involvement of the involved eye, history of headache, jaw claudication, transient monocular visual loss (TMVL), and diplopia were collected. Neuro-ophthalmologic examinations included dilated fundus examination, standard Snellen visual acuity, visual field test, fundus fluorescein angiography, and optical coherence tomography (OCT). Laboratory tests included complete blood count, liver and renal function tests, ESR, C-reactive protein (CRP), rheumatoid factor, anti-streptomycin antibody, and other rheumatology panel tests such as anti-nuclear antibody, anti-extractable nuclear antibodies, and anti-neutrophil cytoplasmic antibody. An infectious disease panel was also included.

### Ultrasound Imaging

Color duplex ultrasonography (CDUS) was performed with MyLab 90 (Esaote, Genova, Italy) to detect ocular arteries [ophthalmic artery (OA), central retinal artery (CRA), and posterior ciliary artery (PCA)]. A high-frequency (6–18 MHz) linear probe was used to detect the superficial cutaneous temporal artery in cooperating patients. The detection site of the superficial cutaneous temporal artery is located in the main trunk of the artery bilaterally in front of the ear.

### Temporal Artery Biopsy

Patients scheduled for superficial temporal artery biopsy were referred to a neurosurgeon. The biopsy was performed in the operating room under local anesthesia, and the severely involved side guided by CDUS was chosen for the biopsy. Long-segment arteries > 3 cm were used for the biopsy to prevent skip lesions (10).

### Statistical Analysis

Descriptive statistics (e.g., means, percentage) were used to summarize the demographic characteristics and clinical features of this case series.

### Ethics Approval and Informed Consent

The Institutional Ethics Review Board of the Eye Ear Nose and Throat Hospital of the Fudan University Shanghai approved all the experimental protocols, and written informed consent was obtained from all participants and/or their legal guardians (KJ-2011-04). The methods were carried out in accordance with the relevant guidelines and regulations. The study was performed in accordance with the Declaration of Helsinki.

## Results

### Demographic and Clinical Features

A total of 15 patients (22 eyes; 10 male, 5 female) were assessed. The mean age was 77.0 ± 8.5 y. Among them, seven patients had bilateral involvement that occurred simultaneously or sequentially. The presenting ophthalmic manifestations included AION (*n* = 12), AION combined with cilioretinal artery occlusion (CLAO) (*n* = 2), and cotton-wool spots (*n* = 1).

Headache was the most common accompanying systemic symptom (*n* = 14, 99.3%) and could be the only presenting symptom. TMVL was the common aura attack (*n* = 13, 86.7%), a detail that was provided by the patient only when asked specifically. Other systemic symptoms included weight loss (*n* = 8, 53.3%), joint pain (*n* = 2, 13.3%), scalp tenderness (*n* = 2), jaw claudication (*n* = 1, 6.7%), and stroke (*n* = 1). None of the patients had diplopia in our case series. There was only one patient with no systemic symptoms.

Visual acuity was very poor, except in patient 3, who presented with only headache without ocular ischemic attack. Most patients having poor visual acuity but ability to perceive light. The visual acuity results are shown in Table 1.

**TABLE 1:** Demographic and clinical characteristics of giant cell arteritis Chinese patients.

No.	Age (y)	Sex	Eye (lateral)	Presenting symptom	HA	TMVL	WL	BCVA	Fundus	ESR (mm/h)	CRP (mg/L)	CDUS	STAU	Biopsy
1	64	M	OS	AION	+	+	−	NLP	Pallid edema	53	77.54	CRA + PCA +	Halo sign Stenosis	P
2	69	M	OD	AION	+	+	−	LP	Pallid edema hemorrhage	33	4.6	CRA + PCA + OA +	Halo sign Segmental stenosis	P
3	66	M	OU	NA	+	−	−	20/40 20/30	Cotton-wool spots	113	80.96	CRA + PCA +	Halo sign Segmental occlusion	P
4	81	M	OD	AION + CLAO	−	+	−	20/100	Pallid edema	65	35.8	CRA + OA +	Halo sign	P
5	76	M	OS	AION	+	+	−	HM	Pallid edema	65	13.1	CRA + PCA + OA +	Normal	N
6	88	F	OS	AION	+	+	−	NLP	Pallid edema	49	8.6	CRA + PCA + OA +	Halo sign Stenosis	N
7	86	M	OD	AION + CLAO	+	+	−	NLP	Pallid edema	22	21.8	PCA + OA +	Halo sign	P
8	79	M	OS	AION	+	+	−	20/400	Pallid edema hemorrhage	50	55	CRA + PCA + OA +	Halo sign Diffuse stenosis	P
9	71	F	OU	AION	+	+	−	NLP NLP	Pallid edema hemorrhage	68	62.8	PCA +	Halo sign	P
10	71	M	OU	AION	+	+	−	LP 20/400	Pallid edema Cotton-wool spots	69	129.5	CRA + PCA + OA +	NA	NA
11	68	M	OD	AION	+	−	−	LP	Pallid edema	27	7.64	PCA +	Halo sign	NA
12	84	F	OU	AION	+	+	−	20/200 20/400	Pallid edema	59	42.8	CRA +	Halo sign	NA
13	90	F	OU	AION	+	+	−	LP NLP	Pallid edema Peripapillary atrophy	63	74	CRA + PCA + OA +	Halo sign Steno sis	NA
14	77	M	OU	AION	+	+	−	NLP NLP	Pallid edema Peripapillary atrophy	90	105	CRA + PCA +	Halo sign	P
15	85	F	OU	AION	+	+	−	NLP NLP	Pallid edema	77	57.3	CRA + PCA + OA +	Halo sign Occlusion	NA

*HA, headache; TMVL, transient monocular visual loss; WL, weight loss; BCVA, best-corrected visual acuity; ESR, erythrocyte sedimentation rate; CRP, C-reactive protein; CDUS, color duplex ultrasonography; STAU, superficial temporal artery ultrasound; M, male; F, female; OS, oculus sinister (left eye); OD, oculus dexter (right eye); OU, oculus uterque (both eyes); AION, anterior ischemic optic neuropathy; CLAO, cilioretinal artery occlusion; HM, hand motion; LP, light perception; NLP, no light perception; CRA, central retinal artery; PCA, posterior ciliary artery; OA, ophthalmic artery; P, positive; N, negative; NA, not available. +In the CDUS column indicates decreased blood flow.*

*CRP normal limit (0–3 mg/L).*

*ESR normal limit (0–15 mm/h).*

**FIGURE 1:**
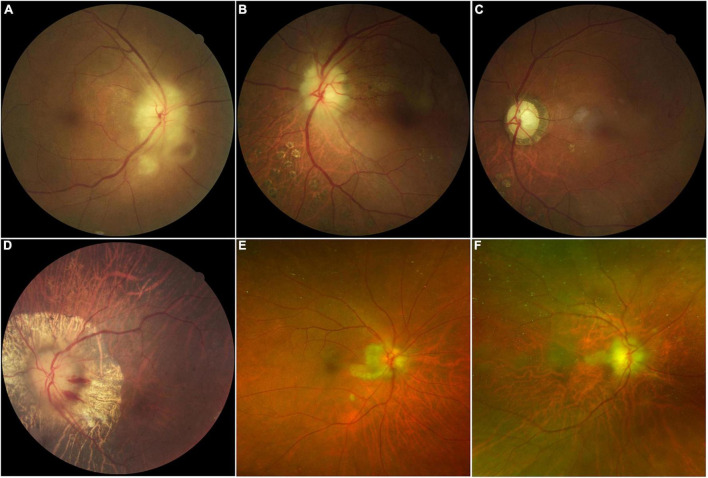
Color fundus photographs in patients with giant cell arteritis showing chalky-white swollen discs **(A,B,D)** with retina exudate and cotton-wool spots **(A)**, late-stage optic atrophy and cupping **(C)**, peripapillary hemorrhage with atrophy **(D)**, and cilioretinal artery occlusion **(B,C,E,F)** are the same eye.

Fundus examination of most AION eyes (15/22 eyes) revealed a classic chalky-white disc swollen due to GCA, and some with peripapillary hemorrhage (4/22 eyes), retinal exudates, and cotton-wool spots (4/22 eyes). CLAO was observed in two patients; both cases were combined with AION. Peripapillary atrophy (6/22 eyes) was also common in patients with severe disease and bilateral involvement. Optic atrophy with cupping was observed in cases of late-stage AION or in eyes with history of previous attack (Figure 1). Details of demographic and clinical features are shown in Table 1.

**FIGURE 2:**
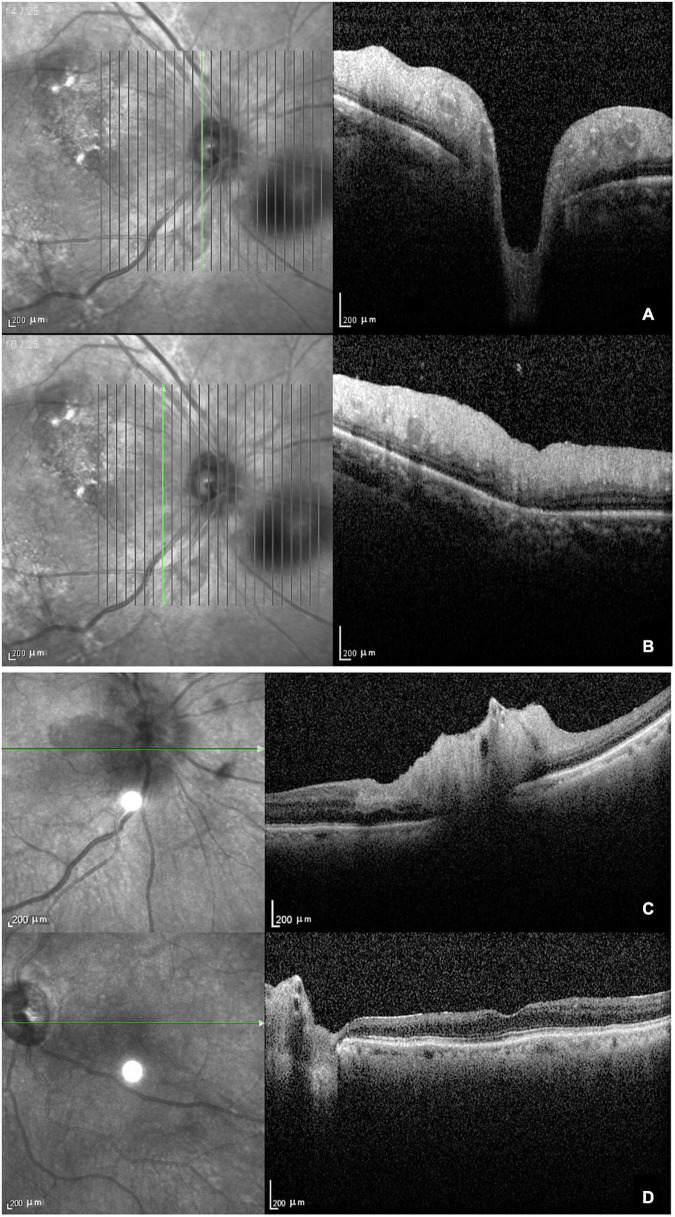
Optical coherence tomography in patients with giant cell arteritis. **(A)** Optic disc edema with a loss of layer structure in the inner retina; **(B)** edematous thickening of the peripapillary retinal nerve fiber layer and ganglion cell layer; **(C)** arteritic anterior ischemic optic neuropathy combined with ciliary vascular obstruction showing edema of the optic disc, thickening, and a loss of layer structure in the inner retina; **(D)** atrophy and thinning of the retinal nerve fiber layer and ganglion cell layer with epiretinal membrane at the late stage.

The main findings of OCT performed in 11 eyes were ischemic retinopathy or neuropathy. Eight eyes were in the acute stage and showed optic disc edema with a loss of layer structure in the inner retina and extreme edematous thickening of the peripapillary retinal nerve fiber layer and ganglion cell layer (Figures 2A,B). In patients with arteritic AION combined with CLAO, OCT showed edema of the optic disc, thickening of the inner retinal nerve fiber layer and ganglion cell layer, and loss of layer structure (Figure 2C). Patients with late-stage bilateral involvement and no light perception had peripapillary atrophy with extremely thin retinal nerve fiber and ganglion cell layers and epiretinal membranes (Figure 2D). Due to their poor systemic condition, only two patients underwent fundus fluorescein angiography, which revealed a delay in optic disc and choroidal artery perfusion.

### Routine Blood Test

The ESR and CRP levels in all patients are listed in Table 1. According to the American College of Rheumatology 1990 criteria for GCA, the ESR was elevated in 11 patients (73.3%), and the CRP was substantially elevated in all 15 patients. The results of the rheumatologic panel were unremarkable, including the rheumatoid, anti-streptomycin antibody, anti-nuclear antibody, and anti-neutrophil cytoplasmic antibody. The complete blood count showed a considerable increase in white blood cell count (*n* = 9) and decrease in red blood cell count and hemoglobin (*n* = 12). The platelet count was elevated in four patients, with increased thrombocytes in nine patients.

### Ultrasonography Imaging

**FIGURE 3:**
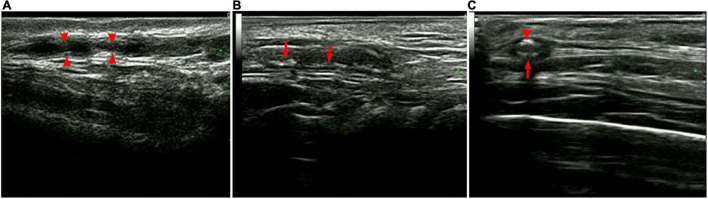
Superficial temporal artery ultrasound. **(A)** Segmental stenosis (arrows); **(B)** segmental thickening with calcification (arrows), and **(C)** hypoechoic halo (large arrow) with calcification (small arrow).

Color duplex ultrasonography (CDUS) detected that almost all patients had varying degrees of ophthalmic ischemic conditions associated with the CRA, PCA, and OA. Among the 15 patients, 14 underwent superficial temporal artery ultrasound with a high-frequency linear probe. The results demonstrated bilateral thickening of the vessel wall with the hypoechoic halo sign, consistent with mural inflammation, in 12 (92.9%) patients. The mural showed rough signals with segmental thickening and stenosis, and some had diffuse stenosis or occlusion. Non-specific calcification was also observed in some individuals (Figure 3). The mean thickness of the intima was 0.49 ± 0.12 mm (0.25-0.75 mm), and the mean vessel diameter was 1.36 ± 0.42 mm (0.74-2.5 mm).

### Magnetic Resonance Imaging Orbit/Brain

**FIGURE 4:**
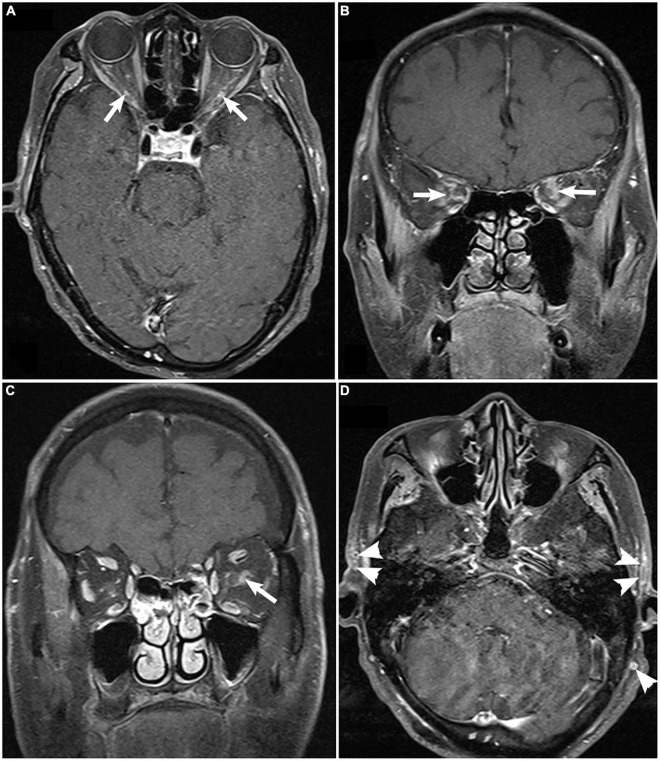
Fat-suppressed contrast-enhanced T1-weighted magnetic resonance imaging of the orbit demonstrating enhancement of bilateral optic nerve sheaths [arrow; **(A)**, axial; **(B)**, coronal], unilateral enhancement of optic nerve sheaths [**(C)**, arrow], and enhancement and thickening superficial temporal artery [**(D)**, arrowhead].

Fat-suppressed contrast-enhanced T1-weighted magnetic resonance imaging (MRI) of the orbit, available for only nine patients demonstrated bilateral optic nerve sheath enhancement. The cranial vessels, including the internal/external carotid arteries and some scalp arteries, showed enhancement of the thickened intima with luminal stenosis or occlusion (Figure 4). Brain MRI showed non-specific diffuse brain atrophy with enlarged ventricles in 11 patients.

### Temporal Artery Biopsy

**FIGURE 5:**
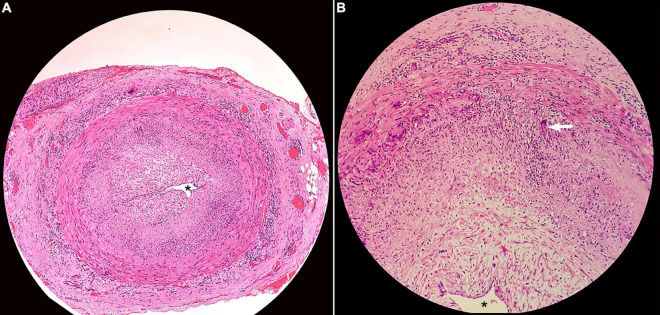
Histopathology of superficial temporal artery biopsy showing **(A)** transmural inflammation of the artery wall, intimal thickening, and near obliteration of the artery lumen (asterisk; H&E × 40); **(B)** multinuclear giant cell (arrow; H&E × 200).

Among the eight patients who underwent temporal artery biopsy, seven (87.5%) demonstrated positive results in terms of classic GCA pathological changes, such as arterial wall inflammation with mononuclear cell infiltration, multinucleated giant cells, and luminal stenosis or occlusion (Figure 5).

### Treatment and Prognosis

All patients underwent cortical steroid treatment after GCA diagnosis. Intravenous methylprednisolone 500 mg/d to 1 g/d were administered to 11 patients and low dose methylprednisolone 120 mg/d or oral prednisone 60 mg/d was prescribed for patients with intolerance due to contraindications and old age. Immunosuppressive agents such as methotrexate were added while oral prednisone was tapered to a low dosage around 5 to 15 mg/d. Tocilizumab was prescribed and well tolerated in two patients. Final visual functions were very poor in most patients, except in one patient who initially presented with only a headache.

## Discussion

The low incidence of GCA among Asians is considered to be associated with the different type of human leukocyte antigen observed in this population (11, 12). In a comparative study by Pereira et al., only one Asian case was reported during an 8-year period (1), whereas in our territory eye center division of neuro-ophthalmology in Shanghai, 15 consecutive cases have been diagnosed within the last 5 years. To date, this is the only case series report on GCA presenting with ocular manifestations in China. It is also a notably large case series report on Asian populations. Our data suggested that ophthalmologists need to remain vigilant about GCA being potentially underdiagnosed among elderly Asian patients with ischemic conditions such as ophthalmic ischemia, central retinal artery occlusion (CRAO)/AION, and AION combined with CLAO; in particular, the latter was highly indicative of a PCA occlusion. There were no cases of branch retinal artery occlusion in this series since they lack the internal elastic lamina (13, 14).

Female predominance has been reported in the literature among both Caucasian and Asian ethnicities (1, 7, 15). The male/female ratio in our case series was 2:1, which was consistent with the ratio found in a small case series with Thai patients (2); both of these case series were composed mainly of patients with AION.

Systemic symptoms such as headache, TMVL, weight loss, and scalp tenderness were common complaints in this study of which headache could be the only presenting symptom. Although TMVL was also very common, it was reported only when the patients were specifically asked. Diplopia and visual hallucinations were not reported by any patient. Unlike Caucasians, Asian patients with GCA usually present with non-specific symptoms such as headache, weight loss, malaise, fatigue, and fever, rather than jaw claudication or double vision. This results in delayed diagnosis by physicians or rheumatologists.

Due to a lack of specific blood biomarkers for the diagnosis of GCA, routine complete blood count, ESR, and CRP are very useful ancillary tests that can quickly distinguish patients with arteritic AION from those with non-arteritic AION. Elevated ESR and CRP levels, combined with anemia, are highly indicative of the necessity for further work-up.

According to the literature, the incidence of visual symptoms in GCA can be as high as 70%, and 20% of patients may experience permanent visual loss (15, 16). In our case series, he visual acuity most tested in the affected eye was light perception, and the outcome was also very poo. The fundus appearance revealed classic chalky-white swelling in the acute stage, and retinal cotton-wool spots might have been an early sign of arterial ischemia. Peripapillary atrophy is another feature in patients with chronic ischemia, which can be observed in the acute stage. The simultaneous presence of AION and CRAO indicates PCA involvement and is highly indicative of GCA causing arteritic AION rather than non-arteritic AION. In addition, AION combined with CRAO/branch retinal artery occlusion, which indicates OA involvement, was not observed in our series.

Due to the considerably low temporal artery biopsy rate in China, GCA is rarely reported by ophthalmologists in this country; therefore, GCA remains underdiagnosed among the AION population, especially in the elderly. Temporal artery ultrasonography, which is non-invasive and cost-effective, is recommended by the European League Against Rheumatism as the first-line study for evaluating large-vessel vasculitis and has been widely utilized in our clinic recently for evaluating high-risk GCA patients (17–19). CDUS can detect the blood flow in ocular arteries, including the OA, CRA, and PCA, and the high-frequency linear probe can detect the superficial cutaneous temporal artery. The hypoechoic halo sign is indicative of inflammatory thickening of the intima with stenosis/occlusion of the lumen; these phenomena are consistent with pathological changes in GCA. According to our data, the halo sign is a highly specific indicator for GCA diagnosis and can also guide the side for biopsy. However, a limitation associated with using ultrasound for detection of temporal artery vasculitis is operator dependence.

MRI is another non-invasive technique for evaluating arteries and determining a diagnosis (17, 20). In addition to the cranial arteries, enhancement of the optic nerve sheath in orbit MRI has been reported as one characteristic of GCA, which was observed in our case series as well (20, 21). Although perineural enhancement is a non-specific phenomenon which can also be observed in infectious and other autoimmune optic neuropathies such as sarcoidosis, MRI on a patient with potential GCA is still crucial to exclude malignant conditions.

The limitations of the study include the short follow-up of patients which limited our ability to obtain long-term effects of immune therapies like Tocilizumab. Furthermore, future research should focus on identifying specific biomarkers of GCA pathogenesis in the Chinese population.

In summary, GCA may be underdiagnosed in elderly Chinese patients with ocular ischemic conditions. The profound visual loss associated with AION/CRAO in elderly patients is highly indicative of GCA in cases of elevated ESR or CRP levels. The hypoechoic halo sign found on ultrasound of the temporal artery is a very sensitive and specific indicator for diagnosis and should be utilized to guide biopsy.

## Data Availability Statement

The datasets used and/or analyzed during the current study are available from the corresponding author on reasonable request.

## Ethics Statement

The studies involving human participants were reviewed and approved by Ethics Review Board of the Eye Ear Nose and Throat Hospital of the Fudan University Shanghai. The patients/participants provided their written informed consent to participate in this study.

## Author Contributions

QC and GT conceived the study. DG and YB performed the biopsy and pathology. WC and JZ participated in the diagnosis. CF and PS collected and interpreted data. QC and CF drafted the manuscript. GT revised the manuscript. XS sponsored by funding. All authors have approved the final manuscript.

## Conflict of Interest

The authors declare that the research was conducted in the absence of any commercial or financial relationships that could be construed as a potential conflict of interest.

## Publisher’s Note

All claims expressed in this article are solely those of the authors and do not necessarily represent those of their affiliated organizations, or those of the publisher, the editors and the reviewers. Any product that may be evaluated in this article, or claim that may be made by its manufacturer, is not guaranteed or endorsed by the publisher.

## Abbreviations

AION: anterior ischemic optic neuropathy
CDUS: color duplex ultrasonography
CLAO: cilioretinal artery occlusion
CRA: central retinal artery
CRAO: central retinal artery occlusion
CRP: C-reactive protein
ESR: erythrocyte sedimentation rate
GCA: Giant cell arteritis
OA: ophthalmic artery
OCT: optical coherence tomography
PCA: posterior ciliary artery
TMVL: transient monocular visual loss.
